# Non‐Physician Contributors to Patient Satisfaction: Insights for Strengthening Philippine Primary Care

**DOI:** 10.1002/jgf2.70095

**Published:** 2026-01-02

**Authors:** Jeff Clyde G. Corpuz

**Affiliations:** ^1^ Department of Theology and Religious Education, College of Liberal Arts De La Salle University Manila Philippines

To the Editor,

Morikawa et al. [1] demonstrate that patient satisfaction in primary care is significantly influenced by the contributions of non‐physician staff, particularly nursing demeanor and care during waiting times. Their multivariable analysis identified nursing staff demeanor (PR 2.06) and waiting time care (PR 1.43) as independent predictors of satisfaction, underscoring that patient experience reflects the cumulative effect of multidisciplinary interactions rather than physician performance alone [1]. These findings offer important reflections for primary care practice, research, and policy development in the Philippines.

In many Philippine primary care settings, patient encounters begin well before the physician consultation. Nurses, midwives, and barangay health workers (BHWs) often serve as the first and most consistent points of contact [2]. The Japanese findings resonate strongly with this context, as Filipino patients interpret warmth, attentiveness, and respectful communication as manifestations of *malasakit*, a culturally embedded expression of care. Emphasizing nursing demeanor affirms the centrality of relational competence in patient experience, particularly in a system where non‐physician staff carry substantial responsibilities for triage, counseling, health education, and emotional support [3].

Similarly, the importance of “waiting time care” aligns with the Philippine reality of long queues in both urban clinics and rural health units. Patient experience is shaped less by the duration of the wait and more by how patients are accompanied during that time. Simple gestures—status updates, brief conversations, blood pressure checks, or reassurance from BHWs—mitigate frustration and foster trust [2].

These insights suggest actionable strategies to enhance patient experience. Structured “waiting time care protocols” in *Konsulta* clinics and rural health units, led by nurses and BHWs, can provide consistent relational support during delays [3]. National training modules on compassionate communication for non‐physician staff may improve relational quality without significant infrastructural investment. Strengthening the role of BHWs as patient navigators acknowledges their unique cultural and relational proximity to communities. Additionally, patient journey mapping can guide improvements in clinic workflows, ensuring that every interaction contributes positively to satisfaction [1].

Okayama proposes that the Longitudinal Integrated Clerkship is an innovative model of community‐based clinical training in which non‐physician staff are trained to provide culturally sensitive care, thereby enhancing patient‐centeredness, fostering trust, and improving overall satisfaction, while also encouraging future healthcare professionals to value team‐based, community‐oriented practice [4]. For General and Family Medicine, these findings suggest future research directions: examining the contributions of BHWs to patient satisfaction and evaluating interventions that integrate relational practices into clinic operations. Policy‐wise, incorporating patient experience metrics—particularly those related to non‐physician interactions—into PhilHealth Konsulta accreditation could incentivize holistic, team‐based care, aligning with the Universal Health Care Act's goal of efficient, human‐centered primary care.

Ultimately, Morikawa et al. highlight a principle long recognized by Filipino communities: healing is inherently relational, and high‐quality care relies on the coordinated engagement of the entire healthcare team. Embedding Filipino relational values into practice and empowering non‐physician staff can enhance patient‐centeredness, cultural responsiveness, and compassion. Integrating these relational practices into training, accreditation, and routine operations offers a pathway toward more holistic, equitable, and effective primary care.

## Author Contributions

**Jeff Clyde G. Corpuz:** conceptualization, investigation, writing – original draft, writing – review and editing.

## Funding

The author has nothing to report.

## Conflicts of Interest

The author declares no conflicts of interest.

## Linked Articles

This article is linked to https://doi.org/10.1002/jgf2.70073.

## Data Availability

The author has nothing to report.

## References

[1] K. Morikawa , T. Ando , S. Tezen , and T. Okada , “Non‐Physician Contributors to Patient Satisfaction in a Japanese Primary Care: A Cross‐Sectional Secondary Analysis of Patient Satisfaction Surveys,” Journal of General and Family Medicine 26, no. 6 (2025): 612–619. 10.1002/jgf2.70073 PMC12585799 41195032

[2] J. C. G. Corpuz , “Communication as Care: Human‐Centered Leadership in Occupational Health and Safety,” Workplace Health & Safety 73 (2025): 540–541, 10.1177/21650799251362384. 40755003

[3] G. W. Goodrich and J. M. Lazenby , “Elements of Patient Satisfaction: An Integrative Review,” Nursing Open 10, no. 3 (2023): 1258–1269. 10.1002/nop2.1437 PMC9912404 36306415

[4] M. Okayama , “Rethinking Community‐Based Clinical Training in Japan: Toward a More Effective Model for Increasing the Number of General Practice Physicians,” Journal of General and Family Medicine 26, no. 5 (2025): 383–384. 10.1002/jgf2.70038 PMC12404172 40904446

